# Multimedia Mixed Reality Interactive Shared Decision-Making Game in Children with Moderate to Severe Atopic Dermatitis, a Pilot Study

**DOI:** 10.3390/children10030574

**Published:** 2023-03-17

**Authors:** Ling-Sai Chang, Ho-Chang Kuo, Jason Jyh-Bin Suen, Pei-Hsin Yang, Chiu-Ping Hou, Hui-Ru Sun, Zon-Min Lee, Ying-Hsien Huang

**Affiliations:** 1 Department of Pediatrics, Kaohsiung Chang Gung Memorial Hospital and Chang Gung University College of Medicine, Kaohsiung 83301, Taiwan; 2 Department of Digital Media Design, I-Shou University, Kaohsiung 83301, Taiwan; 3 Department of Nursing and Pediatric Ward, Chang Gung Memorial Hospital-Kaohsiung Medical Center, Kaohsiung 83301, Taiwan; 4 Department of Pharmacy, Kaohsiung Chang Gung Memorial Hospital, Kaohsiung 83301, Taiwan

**Keywords:** atopic dermatitis, metaverse, multimedia mixed reality game, shared decision making, System Usability Scale

## Abstract

(1) Objective: Atopic dermatitis (AD) is a recurring skin disease that affects children’s daily activities and sleep quality. Due to the limitations of children’s understanding and ability to express themselves, shared decision making (SDM) is often made by guardians, which thus affects the acceptance and effectiveness of children’s treatments. Previous studies have demonstrated that involving both children and parents in decision making may help improve treatment outcomes; thus, we designed a multimedia mixed reality (MR) interactive game of SDM for children with moderate to severe AD. (2) Methods: Research participants included 6–18-year-old patients with moderate to severe AD. This research consisted of the following steps: designing SDM; character setting and visual design; performing games; system modification and optimization; screen editing and dubbing; and user testing and questionnaires by the System Usability Scale (SUS). (3) Results: We completed the SDM design for children with moderate to severe AD. Four different treatments were biologics, oral immune-modulating drugs, phototherapy, and wet wrap. An animated PowerPoint slide showed the AD apple rolling around before treatments and the AD apple sleeping soundly after treatments. Instructions with video teaching for the four different treatments were played, and then, the MR was turned on so that the patients could help the AD apple in the metaverse to undergo these four treatments. A total of 12 moderate to severe AD patients and six control patients used the game, all aged between six and eighteen years old, with an average SUS score of 81.0 and a standard error of 2.1 points. Adjective ratings yielded a rating between good and excellent. The game showed acceptable usability. We found no statistically significant differences in SUS scores between patients with and without moderate to severe AD or between boys and girls nor significant associations between SUS and age or severity. The analysis identified that the two items with the lowest SUS scores were “I think that I would need the support of a technical person to be able to use this product” and “I needed to learn a lot of things before I could get going with this product”. Both of these comments show the limitations of this game. (4) Conclusions: Overall, this study provides the first MR SDM game that has passed the SUS and can be used as an aid in clinical SDM.

## 1. Introduction

Allergic diseases are important chronic diseases in children with many comorbidities [1]. Atopic dermatitis (AD) is a recurring skin disease in which the skin is often chronically inflamed and is often combined with skin infections, thus affecting children’s daily activities, development, sleep quality, behavior, and mood, especially in refractory AD [2,3,4]. Genetic susceptibility, epigenetic modulation, and environmental factors are involved in the pathogenesis of AD [5,6]. Research has also suggested the interaction between filaggrin gene variants and urine phthalate metabolite levels [6]. The phenotypes and clinical presentation of AD are heterogeneous in the pediatric population [7]. Allergen sensitization and filaggrin polymorphisms indicated the risk factors of atopic march in an AD cohort [8]. A database research identified more AD in children with positive immunoglobulin (Ig)E for fruits than negative specific IgE [9]. The role of egg white and its components in AD implied the importance of food in the control of inflammation [10,11]. The inflammatory condition of Kawasaki disease has also been associated with AD [12].

A significant positive correlation between the severity of AD and the quality of life in children has been observed [2]. Up to 85% of caregivers feel that severe AD is not well controlled [13]. Burdens of AD have included missed school and social functioning in school [14]. Effects of AD on sleep disturbances and mental health were found in both the children and their families [13,14]. Clinical trials have suggested that melatonin supplementation has beneficial effects on sleep disturbance in children with AD [15,16]. Exercise behavior and motivation were altered in adolescents with AD [17].

In pediatric allergy care, healthcare professionals need to consider the growing amounts of emerging therapies that have been developed and investigated, mainly targeting type 2 inflammation [18,19]. Considerations in the care of pediatric populations include phototherapy, which is not contraindicated in children but is less frequently used than in adults [3]. Because the long-term safety of phototherapy is unknown, phototherapy should be performed with caution in young children [3]. Another concern is that evidence of the efficacy of labor-intensive wet wraps is of low quality [3,20]. This highlights the importance of education for the care skills of the wet wrap [21,22]. Cyclosporine is approved for severe AD but is less effective than dupilumab, a biologic [23]. The biologic dupilumab has been approved by the Taiwanese Food and Drug Administration for use in children with AD over six years old [24]. Calcineurin inhibitors are approved for children above six months of age. Specialist care is essential for the treatment of severe AD patients [25]. In those who do not respond to conventional treatment, alternative diagnosis and therapy were also considered [24,26]. Hyper IgE syndrome also incorporated other primary immunodeficiency manifestations [27,28]. In Taiwan’s health insurance database, data about the use of traditional Chinese medicine to treat children’s AD were found [26]. Bleach and probiotics are not routinely recommended [24].

The term “shared decision making” (SDM) was first proposed in 1982 in the Common Welfare Program of Patient-Centered Care in the United States to promote mutual respect and communication between doctors and patients. The operational definition, proposed by Charles in 1997, required at least the participation of both the physician and the patient [29]. SDM is becoming particularly important in the perspective of patient engagement and precision medicine. The World Health Organization (WHO) proposed the topic of World Patient Safety Day 2023 as Engaging Patients for Patient Safety. The application of SDM is quite extensive, including the treatment and prevention of diseases [30,31]. Clinical decision support systems have shown the potential for diagnosis and monitoring of allergic diseases [1]. Studies of patient-centered outcomes have demonstrated SDM development in the pediatric field, including for patients with juvenile idiopathic arthritis [32,33].

For successful treatments, motivation and adherence are essential [34]. In the treatment of children’s diseases, due to the limitations of children’s abilities to understand and express themselves, guardians often make SDM, which affects the acceptance and effectiveness of children’s treatments [35]. Furthermore, SDM may be greatly influenced by parent–child interactions, and the research indicates that involving both children and parents in decision making may help improve treatment outcomes. Children have the right to be included in SDM [36]. We hope that children with refractory AD would be included in SDM, with the expectation of improving the understanding, acceptance, and cooperation of children with treatments of the disease. A meta-analysis has suggested that health education can help in the treatment of AD [37]. Online SDM aides already play an important role in childhood asthma [38,39]. Allowing school-aged children to participate in the process of SDM has provided triadic interactions among parents, kids, and medical providers to improve the children’s asthma management [40,41]. The application software showed excellent usability in SDM for parents of febrile infants ≤60 days old [42]. Nevertheless, the impact of pediatric SDM interventions on disease outcomes was unclear [35]. We considered that parents need SDM that is assisted by well-designed software, something children would need even more. Involving both children and parents in decision making has been reported to enhance treatment outcomes related to psychosocial difficulties and functioning [43]. SDM for AD patients was commonly reported in the United States and was associated with better long-term outcomes of symptom control and higher satisfaction in both adults and children [25]. Under the supervision of the Joint Commission of Taiwan, many SDMs for AD have been created in medical institutions. Personalized precision medicine is expected to apply to SDM because of differences in treatment guidelines for children and adults with AD [3,24]. Application of artificial intelligence machine learning algorithms in clinical decision can support children with AD in the future [31].

Gamification, as a playful approach in healthcare, has been suggested to support the treatment of pediatric patients and to promote better user experience in various fields such as pediatric cancer and urology [34,44]. Advancing digital and gamification solutions can overcome longstanding barriers in pediatric patient involvement in SDM. An engagement game for pediatric chronic diseases has been designed for supporting SDM for the empowerment of adolescents [45]. Many fields are rushing to develop technologies related to the metaverse, the keyword showing fusion of the virtual and real worlds. Therefore, we designed a multimedia mixed reality (MR) interactive game of SDM for children with moderate to severe AD and assessed its acceptability and usability by the System Usability Scale (SUS). The findings of the pilot study conducted to test the game hold the promise that our proposed game can be operated clinically. We aim to develop communication across doctors, families, and patients, educate families and patients about the disease, and design children-friendly environments with a playful strategy. The purpose of this study was to build an MR game that supports face-to-face clinical decision aids for children with moderate to severe AD. We hypothesized that children would find the SDM game acceptable.

## 2. Materials and Methods

### 2.1. Participants

We conducted a prospective study. Participants were patients aged 6–18 years who were diagnosed with moderate to severe AD (SCORing AD, SCORAD score ≥ 20 points) in Kaohsiung Chang Gung outpatient clinic or hospital [46]. SCORAD consisted of extent, intensity and subjective symptom scores [17]. The control group testing the game was outpatient or inpatient children aged 6–18 years without moderate or severe AD. The Committee of Kaohsiung Chang Gung Hospital (202100815A3) approved this study. The research began from designing and submitting SDM for review and obtaining approval of the SDM in children with moderate to severe AD.

### 2.2. Design of Mixed Reality Game

**Figure 1:**
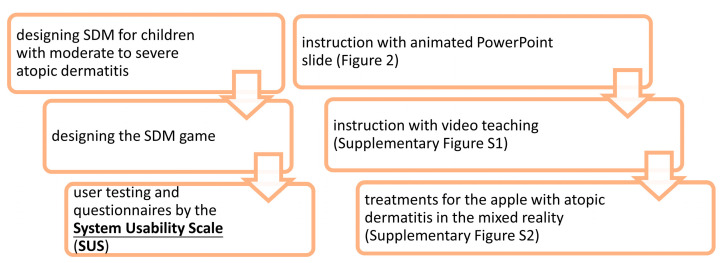
The structure of this prospective study and shared decision-making (SDM) game.

1. Role setting:
  This game sets the AD patient as an apple. The texture of the brush strokes showed skin lesions, which disappeared after receiving treatments.
2. Visual Design:
  The design of this system was multimedia, which consisted of three parts, was carried out using a notebook computer equipped with lenses and speakers, and required specifications that could use MR. The contents of the SDM game are as follows (Figure 1).
  - Animated SDM PowerPoint slides (2 min and 45 s in total):
    The animation showed four different treatments: biologics, immune-modulating oral medication, phototherapy, and wet wrap. The animation showed the apple rolling around before the treatments and sleeping soundly after the treatments. This design aimed to highlight that the treatments by doctors could help improve the condition, thereby increasing compliance with physicians’ orders. Several suggestions were made to improve the user experience of specific components. For children in lower grades who struggled to read, we designed dubbing content that showed the focus of the SDM between doctors and patients. The factors that guided the multimedia interactive game were as follows:
    - Scratching will cause bacteria to get into the inflamed skin, which would worsen the condition.
    - When taking oral medicines, patients should have regular examinations to check their white blood cells and liver and kidney functions.
    - The best medicines right now are biologics [4,23].
    - Patients need to visit the hospital two to three times per week for phototherapy.
    - With doctor’s treatments, patients will get better and better.
    - After watching the instructional video, we are about to enter the metaverse to help the little apple who cannot sleep because of AD.
  - Instructional video (3 min and 50 s in total):
    Demonstration of treatment steps using four dolls with AD. While watching the teaching video, we take out the prepared game props, including cotton swabs, elastics, gauze, lotions, ointments, oral medicines, syringes, and others.
  - Game story design and game method (about 2 min). Open MR and let the patient help the AD apple in the metaverse to undergo the four treatments. This research interface allowed the medical team to freely rotate the angle of the AD apple through the mouse.
3. System modification and optimization;
4. Tests, user tests, and questionnaires:
  For usability testing of this game, we used validated SUS to measure acceptability immediately after playing the game [42]. Questions 1, 3, 5, 7, and 9 were positive questions. The score of each question was subtracted by 1 in order to obtain the score for each question; questions 2, 4, 6, 8, and 10 were negative questions, and the score for each question was subtracted from 5 (Table 1). The total score of questions 1 to 10 was multiplied by 2.5, which provided the total SUS score (Table 1) [42].

**Figure 2:**
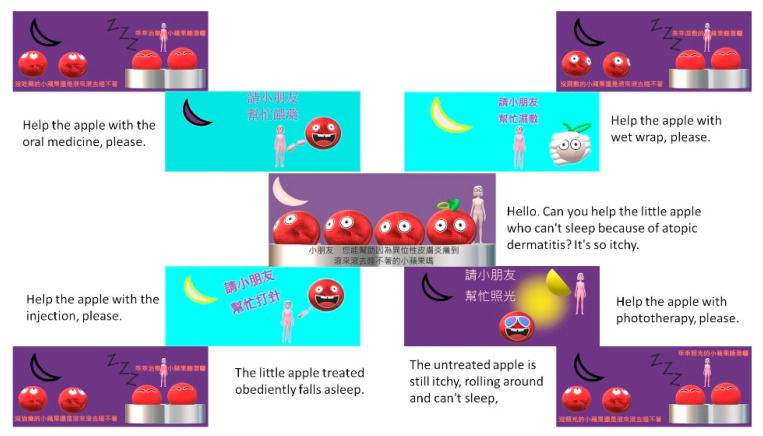
Instruction of shared decision making with animated PowerPoint slide.

### 2.3. Analysis

The analysis used the SPSS statistical software package. First, descriptive statistics were computed and are shown as mean ± standard error. Analyses of variance (ANOVA) with least significant difference (LSD) was used to detect significant differences among various SUS items. The next part of the analysis used Pearson-product moment correlations to exam the relationships between SUS and continuous varieties. Lewis et al. proposed evidence of reliability and suggested that SUS should have sample sizes of at least 12 participants [47].

## 3. Results

We designed SDM for children with moderate to severe AD and then uploaded it after assessing the acceptability, comprehensibility, desirability, and feasibility, and after receiving approval from the organized hospital review board engaging medical and evidence-based medicine professionals for regular use after one round of revision (https://webapp.cgmh.org.tw/cghsdm/PDAS_WRT/Patients/SDM?qid=399; accessed on 6 November 2022). We followed the International Patient Decision Aids Standards [32]. Our research team comprised four pediatric specialists and used consensus meetings to develop the SDM. The committee of the medical quality management center provided an evidence-based state-of-the-art systematic review and meta-analysis for systemic treatments in the management of AD [4]. After uploading to the internet through the hospital’s system on the patient care platform, clinical doctors could issue medical orders for SDM and print QR codes for patients and their families to scan and complete the form for SDM. The resulting SDM included an introduction explaining what AD is and how it is treated and then took users through four steps to help them decide among the refractory AD management options. Step 1 described four treatment options with information on benefits, risks, and practical aspects based on evidence. Step 2 assessed the patient’s and family’s considerations of medical treatments. Respondents were asked to evaluate and score (on a scale of −3 to +3) the importance of each consideration. Step 3 asked whether the patient and family members understood the information provided above. We designed a quiz that included three items on the important information of AD treatments. Finally, Step 4 asked about the selected treatment. Subsequently, a quick patient-reported measure of SDM in clinical encounters was included in the questionnaire to evaluate effectiveness.

The structure of this prospective study and SDM game are illustrated in Figure 1. The developmental process has the potential to implement contextually within children’s experiences and concerns. A children-centered design was used to develop a patient engagement game. We identified the important themes for AD patients regarding their illness and treatments. The identified themes showed the curiosity about treatments and the concern of frequent hospital visits, fear of needles, and perception of comfort levels with treatments in children with AD. We evaluated the draft version of the game. Based on the feedback, we added dubbing content, changed the colors and adjusted the images. Then, animated SDM PowerPoint slides and an instructional video were merged into a film. The total length was 6 min and 32 s. After the introduction (Figure 2) and teaching (Supplementary Figure S1), the patient entered the metaverse, a MR system to treat the AD apple (Supplementary Figure S2). In the MR interactive game (Supplementary Figure S2), AD patients were given their instructions by the doctor using a laptop.

A total of 12 moderate to severe AD patients and six controls tested the game; all participants were aged between six and eighteen years, with an average SUS score of 81.0 and a standard error of 2.1 points (Table 1). The mean age (8.5 ± 0.6 years (SE) years) of the 12 patients with AD was similar to that of the controls (9.7 ± 1.5 years, *p* = 0.494, Table 1). Adjective ratings yielded a rating between good and excellent [48]. The game showed acceptable usability to children [48]. Products that scored by validated SUS in the 80s were good, and products that scored in the 70s were acceptable [48]. We needed the interval to have a 95% confidence and SUS scores ≥ 70; then, we had the power of 0.9996 for total participants (*n* = 18) and 0.967 for AD patients (*n* = 12) by G*Power 3.1.9.7 [49,50,51].

We found no statistically significant difference in SUS scores between controls and patients with moderate to severe AD (*p* = 0.892) or between boys and girls (*p* = 0.328) by Mann–Whitney U test, nor were there significant associations between SUS and age (*p* = 0.905). No significant correlation was uncovered between SUS scores or SCORAD (*p* = 0.426) in patients with moderate to severe AD. The correlations between age and SUS scores in patients with moderate or severe AD were not different (*p* = 0.733). A non-parametric analysis comparing sexes in patients with moderate or severe AD indicated that this difference in SUS scores was nonsignificant (*p* = 0.343). Moderate (SCORAD < 49) and severe (SCORAD ≥ 49) AD accounted for about 41.7% and 58.3%, respectively, of patients. However, there was no significant interaction between SUS scores and moderate or severe AD using a Mann–Whitney U test (*p* = 0.876) [24]. Adolescents aged ≥12 years (*n* = 3) and school-aged children (<12 years, *n* = 15) showed similar SUS scores (*p* = 0.912).

**Figure 3:**
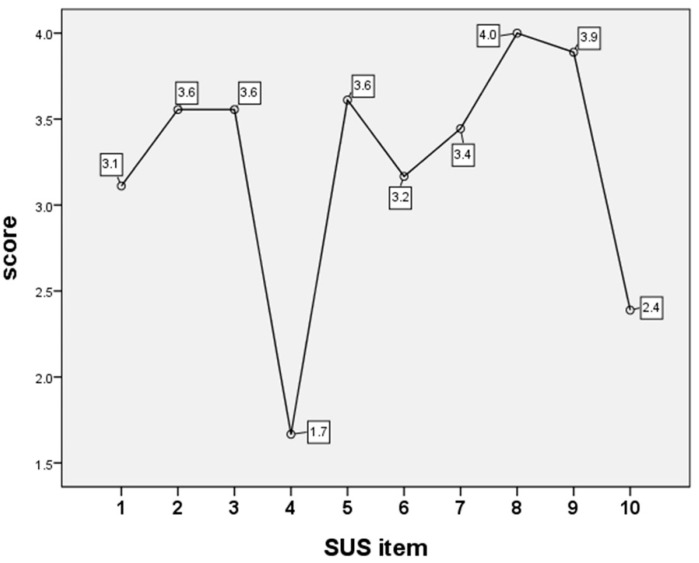
Analysis of variance (ANOVA) analyzed the items of System Usability Scale (SUS) scores shown in Table 1.

ANOVA with LSD analysis identified that the two items with the lowest SUS scores were “I think that I would need the support of a technical person to be able to use this product” and “I needed to learn a lot of things before I could get going with this product” (Figure 3). 

## 4. Discussion

Unlike the previous pediatric study, we first designed the SDM of a specific group and then designed the game according to the SDM [45]. The advantage of following this strategy is that the needs of this specific patient can be better understood, and at the same time, the game can fully express the designed SDM contents. Pediatric patients experience lower rates of SDM engagement compared to adults. In adults, the design of SDM auxiliary tools includes designing SDM first and not designing SDM first. At present, there is no head-to-head comparative analysis of designing SDM first or not [52,53]. In the paragraphs on the methods and results, we can see that the design of SDM and the design of the game each have their own protocols that need to be followed [32,45]. If the SDM is not designed first and then the auxiliary tools are designed, it may confuse the design architectures of the two.

MR is a promising tool and a cost-effective method for patient participation [54]. Our game has the potential to enable children’s involvement. In contrast, virtual reality (VR) requires higher equipment requirements and design costs, which increase the medical burden. Considering children’s need for games and the acceptance of VR, MR is a practical choice. MR is increasingly used in medicine, including medical education, to aid skill learning [54]. The integration of augmented reality (AR), the combination of virtual objects and the real scene into games are acceptable tools for students’ health education [55]. Incorporating AR into a game can improve learning outcomes and emotions [56]. Ten-item SUS out of a maximum of 100 has been applied in many device systems including MR to evaluate usability [48,54]. Internet technological progress has changed the medical field, particularly with regard to SDM and pediatrics [33,42,52,53]. 

The emphasis on patient engagement increased the use of SDM. The SDM tool is a useable and understandable communication tool for clinicians to use with parents, and it is associated with improved knowledge and better perceptions of health risks [39,42]. SDM helps to create more time for communication in pediatric primary care practices so that parents have the opportunity to understand the treatments more deeply and to ask questions about options of treatments. The preferences were discussed, and treatment plans were determined. Face-to-face communication with health care providers has been much more convenient this way. Parents’ open-ended responses suggest that the SDM facilitated communication with their primary care providers, focused attention on AD management, and centralized information on care skills. SDM helps encourage patients receiving more intensive treatments and results in better compliance and favorable outcomes [32,33,57]. An SDM game is a helpful resolution for the underscoring value of providing decision support to children in addition to the parent. Interactive features of the SDM game allowed children to ask questions about the treatments, so that they can obtain first-hand and immediate information. The advantages of digital and audiovisual elements in the SDM game are that they provide varied visual stimuli [52].

Nevertheless, the current shortcomings of the system are “I think that I would need the support of a technical person to be able to use this product” as well as “I needed to learn a lot of things before I could get going with this product”. For example, some difficult medical knowledge, such as biologics in the system, is packaged with the game. The appeal of hands-on roleplaying as a healer for the AD apple is the most attractive and meaningful part of this game; thus, it needs the help of an instructor. Our study and its results were further limited by the small number of participants. While no difference in SUS scores was found between controls and AD or patients with moderate and severe AD, the present study did not assess various emotions to understand the details of playing emotions among controls and patients with moderate and severe AD [56]. Acceptance in health education is possibly due to emotion during the experience of the game [55]. More severe AD is marked by mental health consequences and emotional burdens [14,25].

After development and validation of the game as part of clinical care in this pilot study, future research is needed to investigate the outcomes of SDM and refractory AD. 

## 5. Conclusions

To the best of our knowledge, we are the first to have developed an interactive multimedia MR game of SDM in children with moderate to severe AD. We applied and evaluated the game and demonstrated promising results of usability. As the first decision-making assistance game for children in Taiwan, the content of this game provides not only healthcare knowledge but also a fun experience. Using the research approach, we designed the SDM first and then created an MR game according to our SDM contents.

## Figures and Tables

**Table 1:** System usability scale of multimedia mixed reality interactive game of shared decision-making in participating children.

SUS (Mean ± Standard Error)	Control	Atopic Dermatitis	*p* Value (Mann–Whitney *U* test)	Total
NUMBER	6	12		18
AGE	9.7 ± 1.5	8.5 ± 0.6	0.494	8.9 ± 0.7
SEX (MALE/FEMALE)	4/2	7/5	0.572 by Fisher’s exact test	11/7
SUS SCORE	81.3 ± 2.2	80.8 ± 2.9	0.892	81.0 ± 2.1
1. I’d like to use this system frequently.	3.5 ± 0.2	2.9 ± 0.3	0.213	3.1 ± 0.2
2. I found the system unnecessarily complex.	3.8 ± 0.2	3.4 ± 0.3	0.682	3.6 ± 0.2
3. I found the system easy to use.	3.7 ± 0.3	3.5 ± 0.3	0.682	3.6 ± 0.2
4. I think I’d need support from a technician to be able to use this system.	1.3 ± 0.2	1.8 ± 0.3	0.616	1.7 ± 0.2
5. I found the various functions in this system well integrated.	3.7 ± 0.2	3.6 ± 0.2	0.964	3.6 ± 0.1
6. I thought this system was full of inconsistencies.	3.0 ± 0.6	3.3 ± 0.3	0.820	3.2 ± 0.3
7. I imagine most people could learn to use this system very quickly.	3.3 ± 0.7	3.5 ± 0.2	0.750	3.4 ± 0.3
8. I found the system very cumbersome to use.	4.0 ± 0.0	4.0 ± 0.0	1.000	4.0 ± 0.0
9. I felt very confident using the system.	4.0 ± 0.0	3.8 ± 0.1	0.616	3.9 ± 0.1
10. I had to learn a lot of things before I could begin to use this system.	2.2 ± 0.7	2.5 ± 0.4	0.682	2.4 ± 0.4

SUS, System Usability Scale.

## Data Availability

The datasets analyzed during the current study are available from the corresponding author upon reasonable request.

## Supplementary Materials

The following supporting information can be downloaded at: https://www.mdpi.com/article/10.3390/children10030574/s1, Supplementary Figure S1. Instruction with video teaching; Supplementary Figure S2. Treating the apple with atopic dermatitis with biologics (2A), oral immune-modulating drugs (2B), wet wrap (2C), and phototherapy (2D) using the mixed reality game.
