# Supplementary material for: Multimedia Mixed Reality Interactive Shared Decision-Making Game in Children with Moderate to Severe Atopic Dermatitis, a Pilot Study
Source: Children (Basel). 2023 Mar 17;10(3):574. doi: 10.3390/children10030574 (PMC10047264; doi:10.3390/children10030574)

Supplementary Figure S1. Instruction with video teaching.

This video is not involved in any profit-making model. It is purely for educational purposes. We are not using the characters as a trademark.

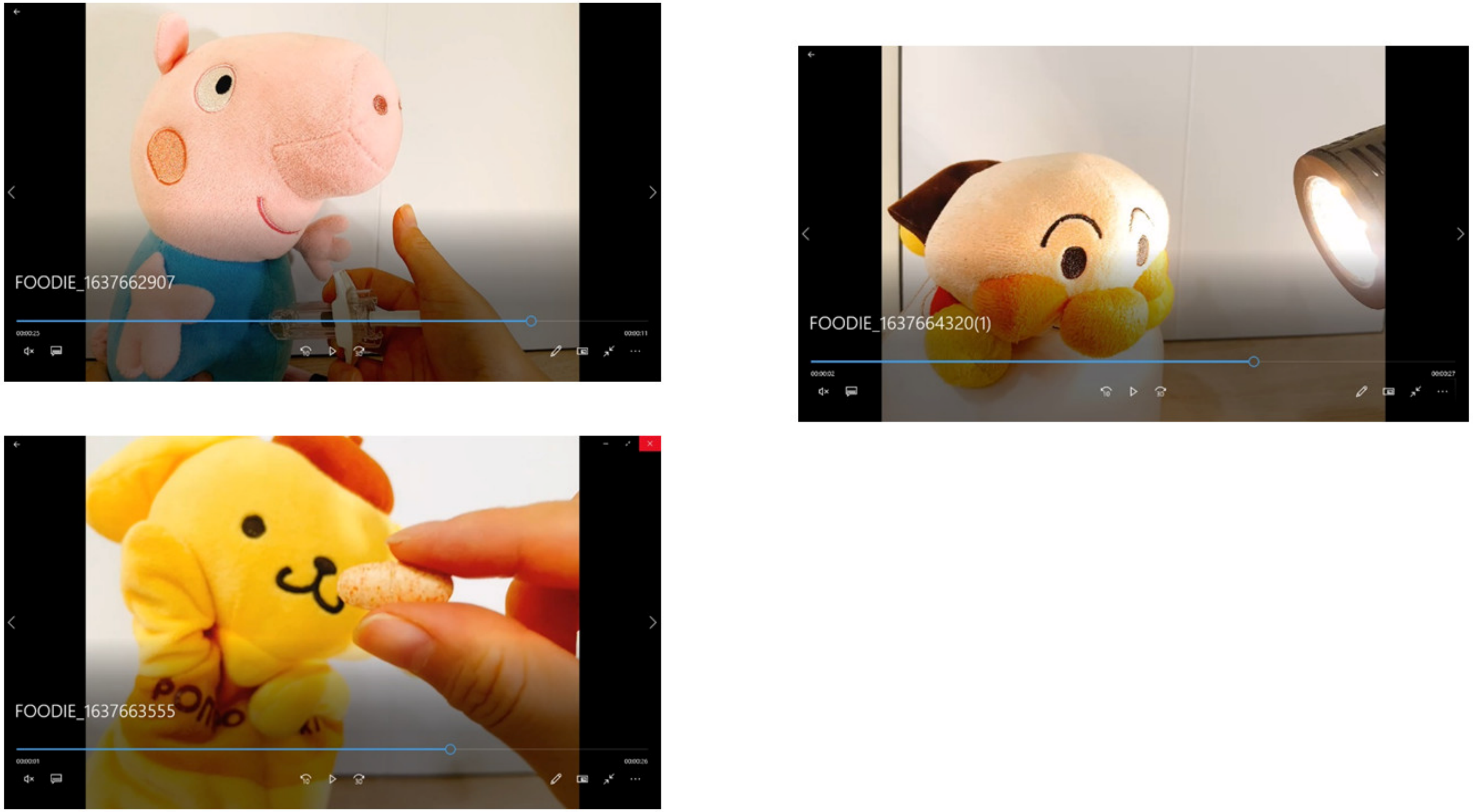

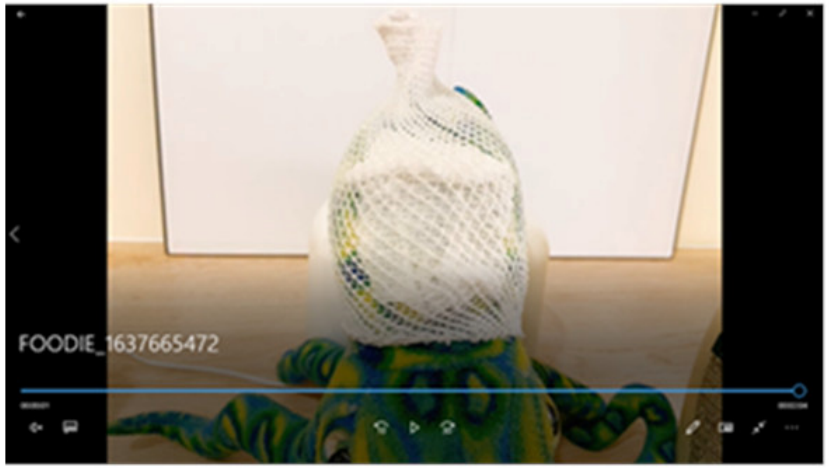
FOODIE_1637665472

Supplementary Figure S2. Treating the apple with atopic dermatitis with biologics (2A), oral immune-modulating drugs (2B), wet wrap (2C), and phototherapy (2D) using the mixed reality game.

Copyright © (2023) (Chang Ling-Sai). All rights of AD apple reserved.

Supplementary Figure S2A.

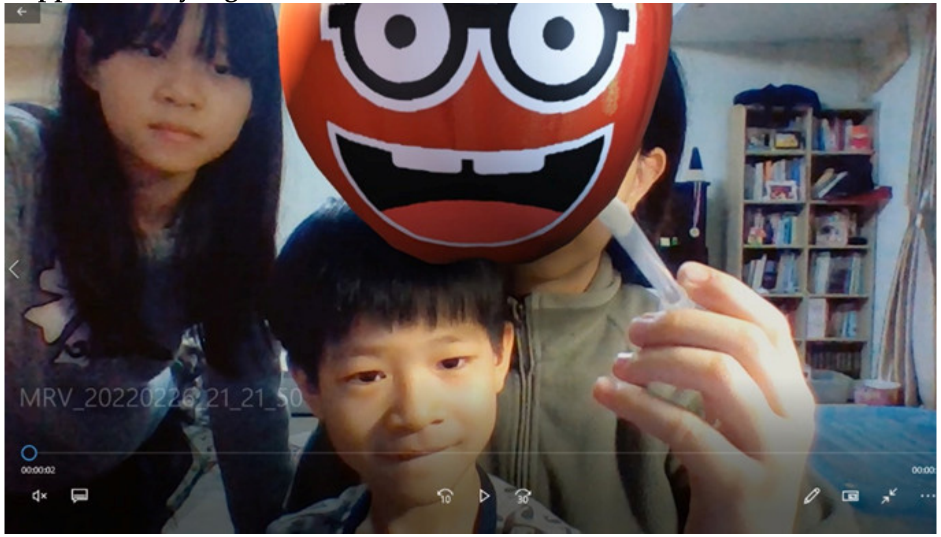

Supplementary Figure S2B.

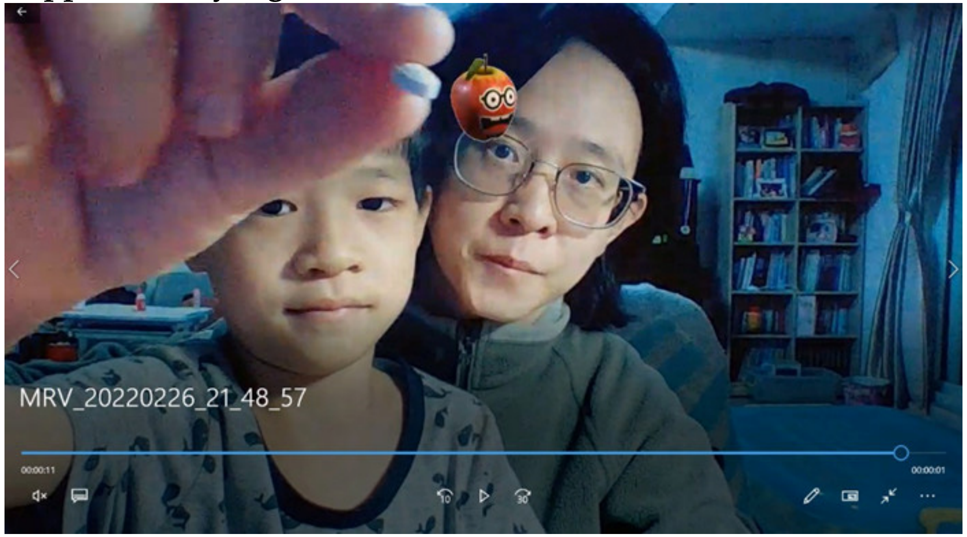

Supplementary Figure S2C.

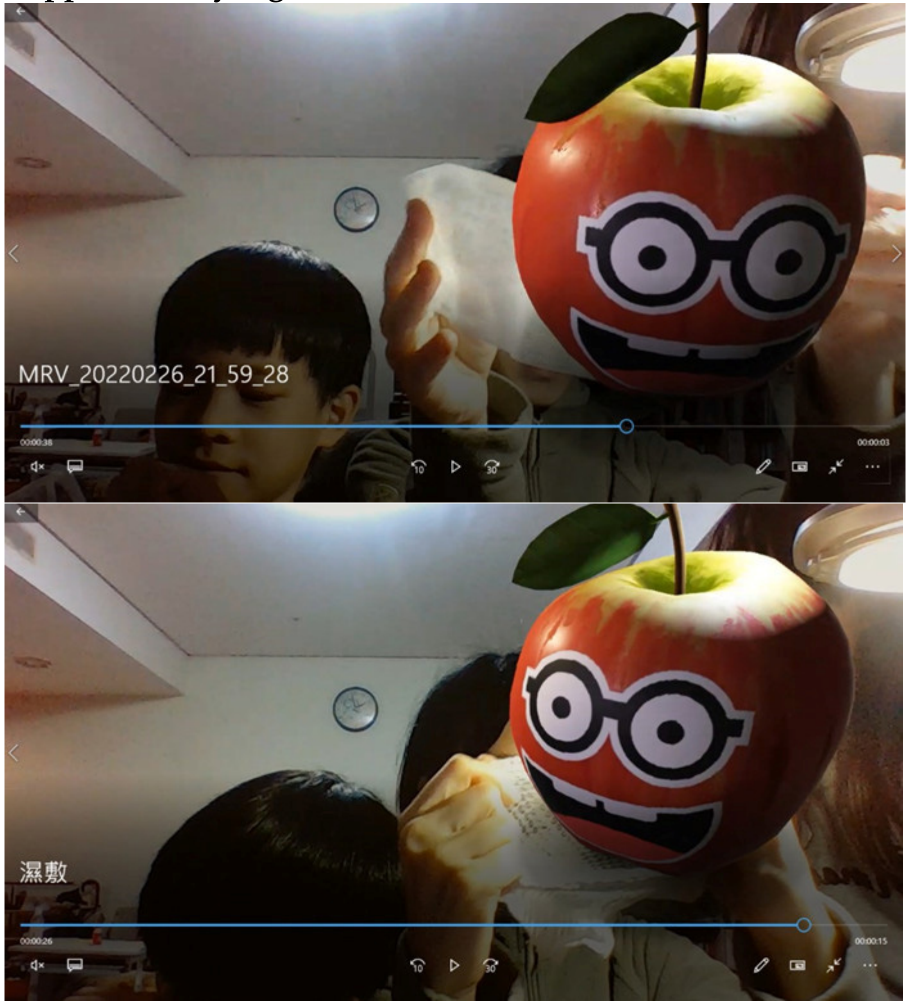

Supplementary Figure S2D.

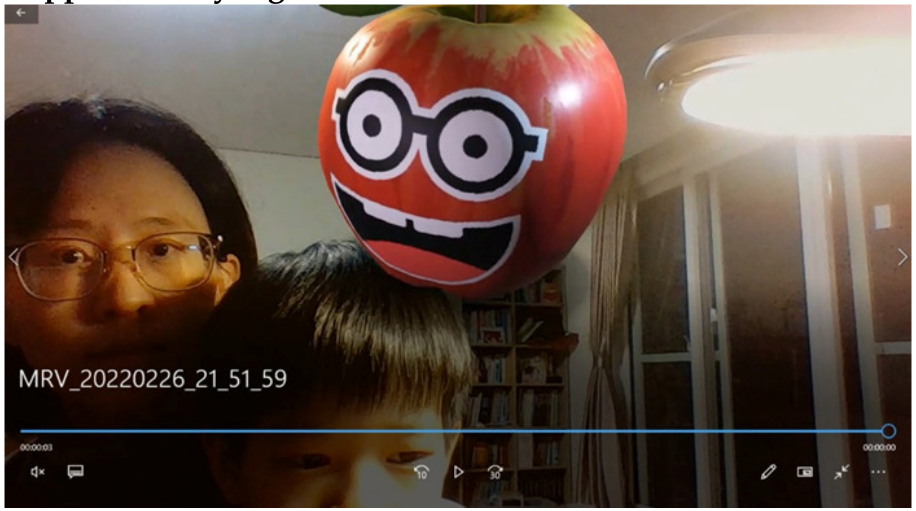

Supplement: Supplementary file 1 [file children-10-00574-s001.zip › children-2231944-supplementary.pdf]
